# Dopaminergic Regulation of Striatal Interneurons in Reward and Addiction: Focus on Alcohol

**DOI:** 10.1155/2015/814567

**Published:** 2015-07-13

**Authors:** Rhona Clarke, Louise Adermark

**Affiliations:** Addiction Biology Unit, Sahlgrenska Academy, Institute of Neuroscience and Physiology, University of Gothenburg, P.O. Box 410, 405 30 Gothenburg, Sweden

## Abstract

Corticobasal ganglia networks coursing through the striatum are key structures for reward-guided behaviors. The ventral striatum (nucleus accumbens (nAc)) and its reciprocal connection with the ventral tegmental area (VTA) represent a primary component of the reward system, but reward-guided learning also involves the dorsal striatum and dopaminergic inputs from the substantia nigra. The majority of neurons in the striatum (>90%) are GABAergic medium spiny neurons (MSNs), but both the input to and the output from these neurons are dynamically controlled by striatal interneurons. Dopamine is a key neurotransmitter in reward and reward-guided learning, and the physiological activity of GABAergic and cholinergic interneurons is regulated by dopaminergic transmission in a complex manner. Here we review the role of striatal interneurons in modulating striatal output during drug reward, with special emphasis on alcohol.

## 1. The Striatal Nucleus

**Figure 1:**
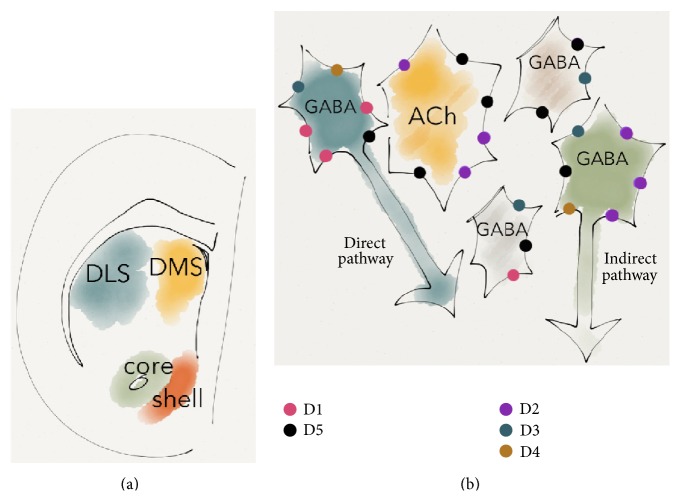
Dopamine receptor expression on striatal neurons. (a) Schematic drawing showing striatal subregions in a rodent coronal brain slice. (b) Dopaminergic receptors are highly expressed on striatal GABAergic (GABA) and cholinergic (ACh) neurons in a pathway and subtype specific manner.

The striatum collects inputs from the entire neocortex and projects to other nuclei in the basal ganglia, ultimately reaching cortical areas implicated in motor planning and execution [1]. Despite apparent similarities in cytoarchitecture, the subregions of the striatum differ with regard to cellular morphology, afferent and efferent circuitry, and receptor localization [2–4]. Based on behavioral studies and subregion-specific extrinsic connections to striatal subregions, the striatal complex is anatomically divided into the ventral striatum (nucleus accumbens (nAc)) and the dorsal striatum (caudate-putamen) [2, 5]. The nAc is a part of the brain reward system and is recruited in pavlovian conditioning [6–8]. This structure can be further subdivided into a shell and core region, where the core bears a greater resemblance to the dorsal striatum, while the shell may be considered a limbic structure and a part of the extended amygdala [9]. The dorsal striatum can be subdivided into the dorsomedial striatum (DMS), which is vital for goal-directed learning, and the dorsolateral striatum (DLS) implicated in stimulus-response learning (Figure 1(a)) [10, 11].

The majority of neurons (>90%) in the rodent striatum are GABAergic medium spiny neurons (MSNs). MSNs can be activated by motor behaviors triggered by both memory-encoded and environmental cues and exhibit highly context-dependent firing patterns [12]. Local microcircuits play an important role in regulating striatal output, with MSNs forming a weak lateral inhibitory network (feedback inhibition), while GABAergic interneurons, despite a lower abundance, exert a more powerful control over striatal excitability (feedforward inhibition) [13]. The striatum also contains cholinergic interneurons, which have been implicated in controlling both glutamatergic and GABAergic transmission onto projecting MSNs [14–16]. In addition, both cholinergic and GABAergic interneurons have been implicated in the induction phase of synaptic plasticity and might thus exert an indirect feedforward control of the excitability of striatal projection neurons [17, 18] The striatal cell population further includes highly interconnected astrocytes that support neuronal activity [19].

## 2. GABAergic Interneurons in the Striatum

The striatal nucleus contains different subtypes of GABAergic interneurons that produce a strong inhibitory postsynaptic potential in MSNs [6, 20–22]. In particular, feedforward inhibition is mediated by parvalbumin-expressing fast-spiking interneurons (FSI) [6, 23]. FSIs have a soma of approximately 16–18 *μ*m in diameter and have moderately branched aspiny dendrites. The local axonal plexus is extremely dense and heavily invested with presynaptic boutons [21]. FSIs comprise roughly 1% of the neuronal population and are distributed in a fashion that shows a ventral to dorsal, medial to lateral, and caudal to rostral gradient of increasing density [4, 24, 25], indicating that these neurons may be especially important in the DLS. FSIs exert powerful monosynaptic inhibition of MSNs through multiple perisomatic synapses and are also themselves coupled by dendritic gap junctions [25]. Striatal FSIs are near-continuously active in awake rodents but often hyperpolarized and silent* in vitro* [26]. The predominant targets of FSIs are MSNs [27], where they synapse on the perikarya [28, 29]. There is a slight preference for direct-pathway MSNs over indirect-pathway MSNs, suggesting a potential mechanism for rapid pathway-specific regulation of striatal output [28, 29].

FSIs play a complex role in coordinating the activity of MSNs, and a deficit in these cells has been observed in dystonia and Tourette syndrome [28, 30]. FSIs filter cortical information, and cross correlation histograms of FSI to MSN pairs in monkey imply that the spikes of MSNs follow those of FSIs and that both are driven by cortical input [22, 31]. Excitatory afferents, primarily from the cortex, form asymmetric synapses on FSIs [25, 32], while symmetric synapses arise from both extrinsic and intrinsic GABAergic (globus pallidus) and dopaminergic (substantia nigra) inputs [33–35]. Pallidostriatal inputs are largely selective for FSIs and increased firing of FSI during choice selection in a simple discrimination task coincides with a decrease in firing of globus pallidus neurons [33, 36], suggesting that pallidostriatal disinhibition may have an important role in timing or coordinating action execution. FSIs do not appear to make functional synaptic connections with other types of GABAergic interneurons or with cholinergic interneurons [37]. But, acetylcholine release from cholinergic interneurons directly depolarizes FSIs via nicotinic receptors, while reducing their influence on MSNs via presynaptic muscarinic receptors on FSI terminals [20].

A second class of GABAergic interneuron comprises the neuropeptide Y (NPY), nitric oxide synthetase, and somatostatin expressing interneurons. These cells have the least dense axonal arborization of all cells in the striatum and receive both cholinergic and dopaminergic inputs [21]. Electrophysiologically these cells are characterized by low threshold calcium spikes and are sometimes termed persistent and low-threshold spiking (LTS) neurons [38]. A third class of GABAergic interneurons is the calretinin expressing interneurons, which colocalize with the calcium binding protein calretinin. These cells are approximately 9–17 *μ*m in diameter and possess few, aspiny dendrites. These cells distribute in a rostrocaudal gradient, with a greater density in the rostral parts of the striatum [39]. Little is still known regarding the electrophysiological properties of these neurons. In addition to these classically defined types, recent studies have revealed novel types of GABAergic interneurons [40, 41]. Some of these interneurons are tyrosine hydroxylase positive, and each type has a unique electrophysiological profile.

## 3. Striatal Cholinergic Interneurons

Cholinergic interneurons are large aspiny cells, which can exceed 40 *μ*M in diameter. In rodent they comprise approximately 1-2% of cells in the striatum [21, 42] but appear to be more frequent in primates [43]. Although few in number, cholinergic interneurons have large axonal arbors, with each cholinergic cell containing about 500000 axonal varicosities [14]. Axon collaterals in the dorsal striatum are largely restricted to the matrix compartment, where they target MSNs, although GABAergic interneurons and other cholinergic interneurons also receive cholinergic input [20, 21, 44]. Cholinergic interneurons are typically tonically active, firing in a slow, regular pattern [14, 45–47]. A pause in cholinergic activity is seen in animals learning stimulus-outcome associations, and GABAergic neurons projecting from the VTA selectively target and inhibit accumbal cholinergic interneurons to enhance stimulus-outcome learning [48]. The synchronous pause in cell firing observed in putative cholinergic interneurons following the presentation of reward or salience-related cues in the striatum, however, depends on input from both nigrostriatal dopaminergic projections and thalamostriatal glutamatergic projections [49–51].

Cholinergic transmission shapes both inhibitory and excitatory influences in the striatum, depending on receptor type and projection target. Striatal cholinergic interneurons modulate striatal activity through the corelease of glutamate and acetylcholine, mediating both glutamate- and acetylcholine-mediated currents in striatal FSIs [52, 53]. In the nAc, optogenetic activation of cholinergic interneurons leads to enhanced activity of MSNs [54], and in the DLS cholinergic interneurons are suggested to exert an excitatory control over D2-expressing MSNs via muscarinic M1 receptors [55]. However, synchronous activation of cholinergic interneurons in the dorsal striatum can also trigger large inhibitory synaptic currents in MSNs by facilitating GABA corelease at dopaminergic terminals [56] and might reduce glutamatergic input to striatal neurons via activation of muscarinic receptors [15, 20, 57]. Ablation of cholinergic interneurons in the DLS produces stereotypies, while no pronounced motor deficits are observed during ablation of cholinergic interneurons in the DMS or during transient inhibition in the nAc [54, 58].

## 4. The Striatal Nucleus and Drug Addiction

Although multiple neurocircuitries are implicated in the rewarding effects of drugs of abuse, the mesocorticolimbic dopamine system is considered the major neurochemical pathway for reward [59, 60]. The reciprocal VTA-nAc circuit is considered the most central part of the reward system [61, 62], and drugs of abuse, including ethanol, have repeatedly been shown to elevate extracellular levels of dopamine in the nAc [63–65]. The nAc shell appears to be particularly important in initial drug actions, with addictive drugs having a greater effect on dopamine release in the shell than in the core [66–68].

Reward associated behavior, however, is an integrative function of corticobasal ganglia networks [5, 69]. Repeated drug intake triggers reorganization of neural circuits, and recruitment of integrative mechanisms within the basal ganglia appears to underlie drug-seeking behaviors associated with addiction [70]. When drug addiction progresses from occasional recreational use to compulsive use, drug-seeking behavior shifts from reward-driven to habit-driven. During this behavioral progression, the control over drug-seeking behavior also appears to shift from nAc to dorsal striatum. In human addicts the dorsal striatum has been implicated in the motivation to obtain the drug and in mechanisms of drug relapse [71, 72], supporting a role for the dorsal striatum during established habitual drug-taking [73]. However, subregions of the dorsal striatum are recruited during different stages of reward and addiction. While the DMS is a central structure during behavioral sensitization to drugs of abuse, the DLS appears to be recruited as addiction develops and the goal-directed control over behavior is replaced by the habit system [73–75]. Importantly, the nAc not only plays a key role during the initial recreational phase of alcohol intake but is also involved during protracted exposure to drugs of abuse. Nicotine-induced effects on accumbal neurotransmission have been shown to persist for months after the last drug-treatment [76], and deep brain stimulation of the nAc has been shown to alleviate alcohol dependency, supporting a role for the nAc also in addiction therapy [77].

## 5. Alcohol and Dopamine in the Striatal Nucleus

Even though fast-scan cyclic voltammetry studies performed in brain slices or anesthetised rodents suggest that ethanol depresses evoked terminal dopamine release in the nAc [78–80],* in vivo* microdialysis conducted on awake and freely moving rodents has repeatedly shown that ethanol increases extrasynaptic dopamine levels in the nAc. This occurs regardless of whether the drug is ingested [81], administered systemically [66, 82, 83], or perfused locally in the nAc [63, 84]. Positron emission tomography (PET) studies have also confirmed that ethanol induces rapid dopamine release in ventral striatum of human subjects [85, 86]. Preclinical research consistently shows that pharmacological manipulations of dopamine transmission in the nAc alter behavioral responses to ethanol [87–90], suggesting that dopamine signaling in the nAc may promote the initiation and maintenance of reward-seeking behaviors. Ethanol-induced dopamine release in the dorsal striatum has not been extensively studied, but enhanced dopamine output has been detected following both systemic and focal administration of ethanol [66, 91, 92]. Importantly, prolonged ethanol intake leads to subregion specific neuroadaptations in striatal subregions [93], supporting the hypothesis that drugs of abuse modulate striatal activity in a subregion selective and integrative manner as the addicted phenotype develops [70, 94].

## 6. Dopaminergic Innervation of the Striatum

Dopamine is a crucial regulator of striatal microcircuitry. Midbrain dopaminergic neurons project in a topographical pattern, with VTA dopaminergic neurons preferentially innervating the nAc, while dopaminergic neurons in the substantia nigra mainly project to the dorsal striatum [2, 3]. Dopamine receptors are present throughout the entire striatal nucleus. The dopamine D1 receptor is positioned on striatonigral MSNs and a subset of GABA interneurons [95–98], while D5 receptors appear to be expressed by all striatal cell populations and in particular by cholinergic interneurons [99–101]. Furthermore, the expression of the D5 receptor is lower in the DLS as compared to the nAc shell [102]. Dopamine D2 receptors are located on striatopallidal MSNs, cholinergic interneurons, and dopaminergic terminals and to some extent on GABAergic and glutamatergic terminals [97, 103–105]. This distribution is also subregion-specific, as D2 receptors are primarily localized on axons and axon terminals in the nAc shell but have a higher prevalence on dendrites and spines in the DLS [102]. Dopamine D3 receptors are expressed on both presynaptic dopamine terminals and postsynaptic GABAergic neurons [58, 106], while D4 receptors are restricted to MSNs [107] (Figure 1(b)).

## 7. Dopaminergic Regulation of GABAergic Interneurons

Dopamine influences striatal interneuron activity via presynaptic and postsynaptic actions and distinct receptor subtypes (Table 1). Overall, FSIs appear to be synchronously affected by drug-induced dopamine release, and while FSI firing rates are positively correlated with drug-induced locomotor activity, MSNs show no consistent relationship [108]. Dopamine receptor blockade has also been shown to modulate the rewarding and aversive properties of nicotine in a manner that correlates with dissociable neuronal activity patterns of FSIs in the nucleus accumbens [109]. Amphetamine and dopamine increase the activity of the majority of FSIs, while dopamine D2 receptor antagonists depress the firing frequency [100, 108]. In particular, FSI activity is enhanced directly through activation of postsynaptic D5 receptors, but dopamine can also affect FSI activity indirectly by simultaneously reducing GABAergic input to FSIs via presynaptic D2 receptors [100, 110]. Enhanced firing of FSIs is also reported in the nAc following withdrawal from cocaine exposure, which might enhance feedforward inhibition [111]. Inhibition of the nigrostriatal circuit causes impaired and poorly timed FSI activity, leading to a consequent weakening of corticostriatal encoding and reduced control of MSNs [112, 113]. 6-Hydroxydopamine injections also reduce the innervation of FSIs to both striatopallidal and striatonigral neurons [114]. The striatal NPY-system also appears to be under tonic influence from dopaminergic afferents [115, 116]. Tyrosine hydroxylase-immunoreactive axons are in synaptic contact with the proximal dendrites and soma of NPY-expressing neurons, and dopaminergic terminals may also express NPY receptors [117, 118]. Dopamine depletion enhances the number of NPY expressing neurons, and repeated administration of methamphetamine enhances preproNPY mRNA expression in a D1-dependent manner [119–121]. Low threshold spiking interneurons are depolarization by dopamine in a D1-like but not D2-like dependent manner [98], while dopamine depletion may lead to a shift from tonic to oscillatory mode, resulting in spontaneous repetitive GABAergic currents in MSNs [122]. Endogenous dopamine has also been shown to influence striatal microcircuitry by negatively regulating the number of striatal TH positive neurons through both direct and indirect mechanisms mediated by multiple dopamine receptor subtypes [123]. Correspondingly, GABA receptors exert a tonic influence over basal dopamine levels in the striatal nucleus [124–127]. In particular, terminal dopamine release is under tonic GABA inhibition, with local GABA_A_ receptor antagonists exerting a stronger influence over dopamine output in nAc as compared to the DLS [124, 125].

## 8. Dopaminergic Regulation of Cholinergic Interneurons

Dopaminergic and cholinergic systems dynamically interact with gate and potentiate sensory inputs to the striatum in a manner that includes presynaptic regulation of neurotransmitter release and postsynaptic effects in target cells [131]. The interaction varies in a manner that depends on the firing frequency of neuronal populations, and the neuronal activity within striatal microcircuits will thus strongly influence how discrete changes in dopamine neuron activity are conveyed. The effect displayed by dopaminergic transmission on cholinergic neurons is highly subregion-specific, and activation of different subtypes of dopamine receptors can elicit opposite effects on acetylcholine release in the striatum [4, 132] (Table 1). Optogenetic activation of dopamine neurons drives a burst-pause firing sequence in cholinergic interneurons in the nAc shell, has mixed actions in the nAc core, and produces a pause in the dorsal striatum [128]. These findings might be explained by regional variations in the corelease of glutamate/GABA from dopaminergic terminals but could also be associated with a heterogeneity in the connectivity between dopaminergic neurons and cholinergic interneurons or may be connected to dopamine receptor expressing inputs that synapse on cholinergic neurons in a subregion-specific manner [56, 102, 128]. Cholinergic interneurons in the shell region of the nAc are also more sensitive to cocaine as compared to neurons located in the core compartment or the DLS [133]. Self-administration of cocaine activates cholinergic interneurons in the nAc, and silencing this drug-induced activity during cocaine exposure prevents cocaine conditioning [54, 133]. However, accumbal acetylcholine has been proposed to dampen excessive dopamine release, and reduced density of cholinergic interneurons in the nAc instead produces a pronounced hyperresponsiveness of the mesolimbic dopamine system and increased sensitivity to cocaine [134, 135].

In the dorsal striatum, stimulation of dopaminergic axons evokes several ionic conductances in cholinergic interneurons, suggesting that dopamine dynamically controls cholinergic tone [130]. Phasic dopamine pauses the firing of cholinergic interneurons but might also produce a delayed excitation [128–130]. Activation of D2-like dopamine receptors on axon terminals reduces synaptic inputs to striatal cholinergic interneurons, while dopamine modulates the excitability of cholinergic interneurons directly through an excitatory D1/D5-mediated postsynaptic mechanism [136]. Local administration of amphetamine or activation of dopamine D2 receptors decreases striatal acetylcholine levels [137, 138], while cholinergic interneurons become more excitable in dopamine-depleted animals [113]. Interestingly, the effect of dopamine depletion on cholinergic interneurons appears to be pathway specific, as 6-hydroxydopamine lesions increase cholinergic innervation of striatopallidal neurons, while the connection to striatonigral neurons is reduced [114].

Similarly, the control of extracellular dopamine levels by endogenous cholinergic activity results from a complex convergence of neurotransmitter/neuromodulator systems. Tonically active cholinergic interneurons modulate dopamine output through activation of both muscarinic and nicotinic cholinergic receptors on dopamine terminals [125, 139–141] and through the corelease of glutamate [142, 143]. Selective activation of cholinergic interneurons enhances accumbal phasic dopamine release, and activation of nicotinic acetylcholine receptors on striatal dopamine terminals contributes to the high probability of dopamine release [144, 145]. Cholinergic control over dopamine transmission depends on the firing frequency, receptor composition, and striatal subregion studied [141, 142]. Distinct populations of nicotinic receptors govern dopamine transmission in the nAc core as compared to the dorsal striatum [146], and while both M2- and M4-muscarinic acetylcholine receptors are necessary for muscarinic regulation of dopamine release in the DLS, only the M4 receptor is necessary in the nAc [140].

## 9. Integrative Function of Interneurons in Ethanol Actions in the Striatum

**Figure 2:**
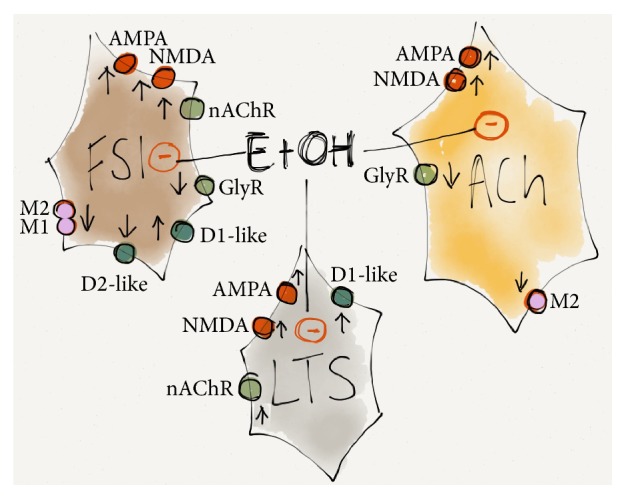
Schematic drawing showing acute effects by ethanol on striatal interneurons. Ethanol exerts a complex modulatory role on striatal interneurons by interacting with multiple receptor systems and signaling pathways, resulting in decreased firing frequency of both GABAergic and cholinergic interneurons, even though FSI activity has been shown to increase in slice recordings performed in the dorsal striatum. Arrows mark the impact on firing frequency caused by activation of the receptor (up/down), while the color marks whether ethanol acts inhibitory (red) or possibly inhibitory (pink) or facilitates (green) receptor activation. In addition, ethanol is presumed to elevate dopamine levels resulting in activation of dopamine receptors (blue), further modulating neuronal activity. See text for further details.

The effects of ethanol on striatal interneurons appear to be highly cell-type specific and region-specific, where decreases in firing rates are shown in cholinergic interneurons and LTSs, while the excitability of FSIs in acutely isolated brain slices is increased in the dorsal striatum [55] (Figure 2). Slice recordings, however, do not always correspond to rate changes recorded* in vivo*, indicating that firing of striatal interneurons is tightly regulated by afferents from other brain regions. Interestingly, electrophysiological recordings performed* in vivo* show a robust decrease in firing frequency in the majority of FSIs in the nAc, indicating subregion specific effects by ethanol [147]. Importantly, both* in vivo* and* in vitro* recordings support the idea that ethanol affects FSIs directly, while MSN activity is indirectly influenced to a greater degree, such as though modulation by the cholinergic tone [55]. The cholinergic tone, on the other hand, is affected by ethanol in a complex manner, leading to changes in firing frequency, transmitter release, and altered receptor affinities [79, 148, 149]. Alcohol consumption selectively promotes C-Fos immunoreactivity in cholinergic interneurons [150], and the density of cholinergic varicosities is reduced during both alcohol consumption and withdrawal [151]. At the same time, electrically evoked release of acetylcholine has been reported to be enhanced in both the dorsal and ventral striatum following a period of alcohol consumption [152]. Systemic administration of ethanol in naïve rodents also increases acetylcholine release in the VTA, but it is not clear if a similar enhancement occurs locally in the striatal nucleus [153, 154]. Electrophysiological slice recordings support a role for cholinergic interneurons in mediating ethanol-induced effects on striatal neurotransmission [55, 155], but nicotinic acetylcholine receptors do not appear to play a crucial role in regulating ethanol-effects on accumbal GABAergic neurons [156]. Importantly, the involvement of cholinergic interneurons in alcohol reward is further supported by the crucial role of glycine receptors, expressed by striatal interneurons, in regulating ethanol-induced dopamine elevations and alcohol consumption in rat [63, 157–162]. NPY also appears to have a modulatory effect on ethanol consumption, and an increased population of NPY-immunoreactive cells and fibers has been reported in rats conditioned to self-administer ethanol [151, 163].

## 10. Conclusion

Even though they comprise less than 5% of the neuronal population in the rodent striatum, interneurons play a major role in regulating the response to drugs of abuse, including ethanol. Both cholinergic interneurons and GABAergic interneurons express receptors that are crucial for the rewarding properties of drugs of abuse, and altered activity of striatal interneurons might be a crucial component during the formation of behavioral adaptations associated with addiction and the motivation to consume the drug. Defining the regulation of striatal interneurons is thus an important step in understanding the physiology of the basal ganglia and in developing pharmacological compounds that are able to selectively target a specific neuronal function.

## Figures and Tables

**Table 1:** Dopaminergic regulation of striatal interneurons.

Neuron type	Subregion	Manipulation	Effect on firing rate	Reference
FSI	nAc	Nicotine + dopamine receptor antagonist/*in vivo *	↓	[109]
FSI	nAc	Cocaine withdrawal	↑	[111]
FSI	Striatum	Amphetamine/*in vivo *	↑	[108]
FSI	Striatum	Dopamine in D1KO via D5R	↑	[100]
FSI	Striatum	Dopamine, cocaine, via D1R activation	↑	[110]
FSI	Striatum	D2R activation	↓	[100, 108]
FSI	Striatum	Impaired nigrostriatal connectivity	↓	[112, 113]
LTS	Striatum	Dopamine via D1-type R	↑	[98]
Cholinergic interneurons	nAc	Optogenetic activation of dopamine neurons	↑	[128, 129]
Cholinergic interneurons	Striatum	Optogenetic activation of dopamine neurons	↓	[128, 130]
Cholinergic interneurons	Striatum	D2R activation	↓	[100]
Cholinergic interneurons	Striatum	Dopamine depletion	↑	[113]

## References

[1] Flaherty A. W., Graybiel A. M. (1994). Input-output organization of the sensorimotor striatum in the squirrel monkey. *The Journal of Neuroscience*.

[2] Voorn P., Vanderschuren L. J. M. J., Groenewegen H. J., Robbins T. W., Pennartz C. M. A. (2004). Putting a spin on the dorsal-ventral divide of the striatum. *Trends in Neurosciences*.

[3] Ikemoto S. (2007). Dopamine reward circuitry: two projection systems from the ventral midbrain to the nucleus accumbens-olfactory tubercle complex. *Brain Research Reviews*.

[4] Gerfen C. R. (1992). The neostriatal mosaic: multiple levels of compartmental organization. *Trends in Neurosciences*.

[5] Yin H. H., Ostlund S. B., Balleine B. W. (2008). Reward-guided learning beyond dopamine in the nucleus accumbens: the integrative functions of cortico-basal ganglia networks. *European Journal of Neuroscience*.

[6] Koós T., Tepper J. M. (1999). Inhibitory control of neostriatal projection neurons by GABAergic interneurons. *Nature Neuroscience*.

[7] Löf E., Ericson M., Stomberg R., Söderpalm B. (2007). Characterization of ethanol-induced dopamine elevation in the rat nucleus accumbens. *European Journal of Pharmacology*.

[8] Sunsay C., Rebec G. V. (2014). Extinction and reinstatement of phasic dopamine signals in the nucleus accumbens core during Pavlovian conditioning. *Behavioral Neuroscience*.

[9] Záborszky L., Alheid G. F., Beinfeld M. C., Eiden L. E., Heimer L., Palkovits M. (1985). Cholecystokinin innervation of the ventral striatum: a morphological and radioimmunological study. *Neuroscience*.

[10] Yin H. H., Knowlton B. J., Balleine B. W. (2006). Inactivation of dorsolateral striatum enhances sensitivity to changes in the action-outcome contingency in instrumental conditioning. *Behavioural Brain Research*.

[11] Yin H. H., Ostlund S. B., Knowlton B. J., Balleine B. W. (2005). The role of the dorsomedial striatum in instrumental conditioning. *European Journal of Neuroscience*.

[12] Hikosaka O., Sakamoto M., Usui S. (1989). Functional properties of monkey caudate neurons. III. Activities related to expectation of target and reward. *Journal of Neurophysiology*.

[13] Tepper J. M., Koós T., Wilson C. J. (2004). GABAergic microcircuits in the neostriatum. *Trends in Neurosciences*.

[14] Zhou F.-M., Wilson C. J., Dani J. A. (2002). Cholinergic interneuron characteristics and nicotinic properties in the striatum. *Journal of Neurobiology*.

[15] Pakhotin P., Bracci E. (2007). Cholinergic interneurons control the excitatory input to the striatum. *Journal of Neuroscience*.

[16] Sullivan M. A., Chen H., Morikawa H. (2008). Recurrent inhibitory network among striatal cholinergic interneurons. *Journal of Neuroscience*.

[17] Centonze D., Gubellini P., Bernardi G., Calabresi P. (1999). Permissive role of interneurons in corticostriatal synaptic plasticity. *Brain Research Reviews*.

[18] Adermark L. (2011). Modulation of endocannabinoid-mediated long-lasting disinhibition of striatal output by cholinergic interneurons. *Neuropharmacology*.

[19] Adermark L., Lovinger D. M. (2008). Electrophysiological properties and gap junction coupling of striatal astrocytes. *Neurochemistry International*.

[20] Koós T., Tepper J. M. (2002). Dual cholinergic control of fast-spiking interneurons in the neostriatum. *Journal of Neuroscience*.

[21] Tepper J. M., Bolam J. P. (2004). Functional diversity and specificity of neostriatal interneurons. *Current Opinion in Neurobiology*.

[22] Mallet N., Le Moine C., Charpier S., Gonon F. (2005). Feedforward inhibition of projection neurons by fast-spiking GABA interneurons in the rat striatum in vivo. *The Journal of Neuroscience*.

[23] Orduz D., Bischop D. P., Schwaller B., Schiffmann S. N., Gall D. (2013). Parvalbumin tunes spike-timing and efferent short-term plasticity in striatal fast spiking interneurons. *Journal of Physiology*.

[24] Luk K. C., Sadikot A. F. (2001). GABA promotes survival but not proliferation of parvalbumin-immunoreactive interneurons in rodent neostriatum: an *in vivo* study with stereology. *Neuroscience*.

[25] Kita H., Kosaka T., Heizmann C. W. (1990). Parvalbumin-immunoreactive neurons in the rat neostriatum: a light and electron microscopic study. *Brain Research*.

[26] Berke J. D. (2011). Functional properties of striatal fast-spiking interneurons. *Frontiers in Systems Neuroscience*.

[27] Bennett B. D., Bolam J. P. (1994). Synaptic input and output of parvalbumin-immunoreactive neurons in the neostriatum of the rat. *Neuroscience*.

[28] Gittis A. H., Nelson A. B., Thwin M. T., Palop J. J., Kreitzer A. C. (2010). Distinct roles of GABAergic interneurons in the regulation of striatal output pathways. *The Journal of Neuroscience*.

[29] Planert H., Szydlowski S. N., Hjorth J. J. J., Grillner S., Silberberg G. (2010). Dynamics of synaptic transmission between fast-spiking interneurons and striatal projection neurons of the direct and indirect pathways. *Journal of Neuroscience*.

[30] Kreitzer A. C., Berke J. D. (2011). Investigating striatal function through cell-type-specific manipulations. *Neuroscience*.

[31] Adler A., Katabi S., Finkes I., Prut Y., Bergman H. (2013). Different correlation patterns of cholinergic and GABAergic interneurons with striatal projection neurons. *Frontiers in Systems Neuroscience*.

[32] Kita H. (1993). GABAergic circuits of the striatum. *Progress in Brain Research*.

[33] Bevan M. D. (1998). Selective innervation of neostriatal interneurons by a subclass of neuron in the globus pallidus of the rat. *Journal of Neuroscience*.

[34] Kubota Y., Inagaki S., Kito S., Wu J.-Y. (1987). Dopaminergic axons directly make synapses with GABAergic neurons in the rat neostriatum. *Brain Research*.

[35] Kubota Y., Inagaki S., Shimada S., Kito S., Eckenstein F., Tohyama M. (1987). Neostriatal cholinergic neurons receive direct synaptic inputs from dopaminergic axons. *Brain Research*.

[36] Gage G. J., Stoetzner C. R., Wiltschko A. B., Berke J. D. (2010). Selective activation of striatal fast-spiking interneurons during choice execution. *Neuron*.

[37] Szydlowski S. N., Pollak Dorocic I., Planert H., Carlén M., Meletis K., Silberberg G. (2013). Target selectivity of feedforward inhibition by striatal fast-spiking interneurons. *Journal of Neuroscience*.

[38] Kawaguchi Y., Wilson C. J., Augood S. J., Emson P. C. (1995). Striatal interneurones: chemical, physiological and morphological characterization. *Trends in Neurosciences*.

[39] Bennett B. D., Bolam J. P. (1993). Characterization of calretinin-immunoreactive structures in the striatum of the rat. *Brain Research*.

[40] Ibáñez-Sandoval O., Tecuapetla F., Unal B., Shah F., Koós T., Tepper J. M. (2010). Electrophysiological and morphological characteristics and synaptic connectivity of tyrosine hydroxylase-expressing neurons in adult mouse striatum. *The Journal of Neuroscience*.

[41] Munoz-Manchado A. B., Foldi C., Szydlowski S. (2014). Novel striatal GABAergic interneuron populations labeled in the 5HT3aEGFP mouse. *Cerebral Cortex*.

[42] Kreitzer A. C. (2009). Physiology and pharmacology of striatal neurons. *Annual Review of Neuroscience*.

[43] Graveland G. A., Difiglia M. (1985). The frequency and distribution of medium-sized neurons with indented nuclei in the primate and rodent neostriatum. *Brain Research*.

[44] Lenz J. D., Lobo M. K. (2013). Optogenetic insights into striatal function and behavior. *Behavioural Brain Research*.

[45] Wilson C. J., Chang H. T., Kitai S. T. (1990). Firing patterns and synaptic potentials of identified giant aspiny interneurons in the rat neostriatum. *The Journal of Neuroscience*.

[46] Bennett B. D., Wilson C. J. (1999). Spontaneous activity of neostriatal cholinergic interneurons in vitro. *The Journal of Neuroscience*.

[47] Zhou F.-M., Wilson C., Dani J. A. (2003). Muscarinic and nicotinic cholinergic mechanisms in the mesostriatal dopamine systems. *Neuroscientist*.

[48] Brown M. T. C., Tan K. R., O'Connor E. C., Nikonenko I., Muller D., Lüscher C. (2012). Ventral tegmental area GABA projections pause accumbal cholinergic interneurons to enhance associative learning. *Nature*.

[49] Reynolds J. N. J., Wickens J. R. (2004). The corticostriatal input to giant aspiny interneurons in the rat: a candidate pathway for synchronising the response to reward-related cues. *Brain Research*.

[50] Aosaki T., Graybiel A. M., Kimura M. (1994). Effect of the nigrostriatal dopamine system on acquired neural responses in the striatum of behaving monkeys. *Science*.

[51] Aosaki T., Tsubokawa H., Ishida A., Watanabe K., Graybiel A. M., Kimura M. (1994). Responses of tonically active neurons in the primate's striatum undergo systematic changes during behavioral sensorimotor conditioning. *The Journal of Neuroscience*.

[52] Nelson A. B., Bussert T. G., Kreitzer A. C., Seal R. P. (2014). Striatal cholinergic neurotransmission requires VGLUT3. *Journal of Neuroscience*.

[53] Hughes J. R., Keely J., Naud S. (2004). Shape of the relapse curve and long-term abstinence among untreated smokers. *Addiction*.

[54] Witten I. B., Lin S.-C., Brodsky M. (2010). Cholinergic interneurons control local circuit activity and cocaine conditioning. *Science*.

[55] Blomeley C. P., Cains S., Smith R., Bracci E. (2011). Ethanol affects striatal interneurons directly and projection neurons through a reduction in cholinergic tone. *Neuropsychopharmacology*.

[56] Nelson A. B., Hammack N., Yang C. F., Shah N. M., Seal R. P., Kreitzer A. C. (2014). Striatal cholinergic interneurons Drive GABA release from dopamine terminals. *Neuron*.

[57] Ebihara K., Yamamoto K., Ueda K., Koshikawa N., Kobayashi M. (2013). Cholinergic interneurons suppress action potential initiation of medium spiny neurons in rat nucleus accumbens shell. *Neuroscience*.

[58] Xi Z.-X., Gardner E. L. (2007). Pharmacological actions of NGB 2904, a selective dopamine D3 receptor antagonist, in animal models of drug addiction. *CNS Drug Reviews*.

[59] Wise R. A., Rompre P. P. (1989). Brain dopamine and reward. *Annual Review of Psychology*.

[60] Engel J., Carlsson A. (1977). Catecholamines and behavior. *Current Developments in Psychopharmacology*.

[61] Koob G. F. (1992). Neural mechanisms of drug reinforcement. *Annals of the New York Academy of Sciences*.

[62] Koob G. F. (1992). Drugs of abuse: anatomy, pharmacology and function of reward pathways. *Trends in Pharmacological Sciences*.

[63] Adermark L., Clarke R. B. C., Olsson T., Hansson E., Söderpalm B., Ericson M. (2011). Implications for glycine receptors and astrocytes in ethanol-induced elevation of dopamine levels in the nucleus accumbens. *Addiction Biology*.

[64] Söderpalm B., Löf E., Ericson M. (2009). Mechanistic studies of ethanol's interaction with the mesolimbic dopamine reward system. *Pharmacopsychiatry*.

[65] Koob G. F., Rassnick S., Heinrichs S., Weiss F. (1994). Alcohol, the reward system and dependence. *Toward a Molecular Basis of Alcohol Use and Abuse*.

[66] Di Chiara G., Imperato A. (1988). Drugs abused by humans preferentially increase synaptic dopamine concentrations in the mesolimbic system of freely moving rats. *Proceedings of the National Academy of Sciences of the United States of America*.

[67] Sellings L. H. L., Clarke P. B. S. (2003). Segregation of amphetamine reward and locomotor stimulation between nucleus accumbens medial shell and core. *Journal of Neuroscience*.

[68] McBride W. J., Murphy J. M., Ikemoto S. (1999). Localization of brain reinforcement mechanisms: intracranial self-administration and intracranial place-conditioning studies. *Behavioural Brain Research*.

[69] Belin-Rauscent A., Everitt B. J., Belin D. (2012). Intrastriatal shifts mediate the transition from drug-seeking actions to habits. *Biological Psychiatry*.

[70] Belin D., Everitt B. J. (2008). Cocaine seeking habits depend upon dopamine-dependent serial connectivity linking the ventral with the dorsal striatum. *Neuron*.

[71] Volkow N. D., Wang G.-J., Telang F. (2006). Cocaine cues and dopamine in dorsal striatum: mechanism of craving in cocaine addiction. *Journal of Neuroscience*.

[72] Volkow N. D., Fowler J. S., Wang G.-J., Swanson J. M., Telang F. (2007). Dopamine in drug abuse and addiction: results of imaging studies and treatment implications. *Archives of Neurology*.

[73] Gerdeman G. L., Partridge J. G., Lupica C. R., Lovinger D. M. (2003). It could be habit forming: drugs of abuse and striatal synaptic plasticity. *Trends in Neurosciences*.

[74] Durieux P. F., Schiffmann S. N., D'Exaerde A. D. K. (2012). Differential regulation of motor control and response to dopaminergic drugs by D1R and D2R neurons in distinct dorsal striatum subregions. *The EMBO Journal*.

[75] Yin H. H., Mulcare S. P., Hilário M. R. F. (2009). Dynamic reorganization of striatal circuits during the acquisition and consolidation of a skill. *Nature Neuroscience*.

[76] Morud J., Adermark L., Perez-Alcazar M., Ericson M., Söderpalm B. (2015). Nicotine produces chronic behavioral sensitization with changes in accumbal neurotransmission and increased sensitivity to re-exposure. *Addiction Biology*.

[77] Kuhn J., Lenartz D., Huff W. (2007). Remission of alcohol dependency following deep brain stimulation of the nucleus accumbens: valuable therapeutic implications?. *Journal of Neurology, Neurosurgery and Psychiatry*.

[78] Budygin E. A., Phillips P. E. M., Wightman R. M., Jone S. R. (2001). Terminal effects of ethanol on dopamine dynamics in rat nucleus accumbens: an in vitro voltammetric study. *Synapse*.

[79] Schilaty N. D., Hedges D. M., Jang E. Y. (2014). Acute ethanol inhibits dopamine release in the nucleus accumbens via *α*6 nicotinic acetylcholine receptors. *Journal of Pharmacology and Experimental Therapeutics*.

[80] Yorgason J. T., Ferris M. J., Steffensen S. C., Jones S. R. (2014). Frequency-dependent effects of ethanol on dopamine release in the nucleus accumbens. *Alcoholism: Clinical and Experimental Research*.

[81] Ericson M., Blomqvist O., Engel J. A., Söderpalm B. (1998). Voluntary ethanol intake in the rat and the associated accumbal dopamine overflow are blocked by ventral tegmental mecamylamine. *European Journal of Pharmacology*.

[82] Campbell A. D., McBride W. J. (1995). Serotonin-3 receptor and ethanol-stimulated dopamine release in the nucleus accumbens. *Pharmacology, Biochemistry and Behavior*.

[83] Blomqvist O., Engel J. A., Nissbrandt H., Söderpalm B. (1993). The mesolimbic dopamine-activating properties of ethanol are antagonized by mecamylamine. *European Journal of Pharmacology*.

[84] Yoshimoto K., McBride W. J., Lumeng L., Li T.-K. (1992). Alcohol stimulates the release of dopamine and serotonin in the nucleus accumbens. *Alcohol*.

[85] Aalto S., Ingman K., Alakurtti K. (2015). Intravenous ethanol increases dopamine release in the ventral striatum in humans: PET study using bolus-plus-infusion administration of [^11^C]raclopride. *Journal of Cerebral Blood Flow and Metabolism*.

[86] Boileau I., Assaad J.-M., Pihl R. O. (2003). Alcohol promotes dopamine release in the human nucleus accumbens. *Synapse*.

[87] Abrahao K. P., Quadros I. M. H., Andrade A. L. M., Souza-Formigoni M. L. O. (2012). Accumbal dopamine D2 receptor function is associated with individual variability in ethanol behavioral sensitization. *Neuropharmacology*.

[88] Adamantidis A. R., Tsai H.-C., Boutrel B. (2011). Optogenetic interrogation of dopaminergic modulation of the multiple phases of reward-seeking behavior. *The Journal of Neuroscience*.

[89] Bahi A., Dreyer J.-L. (2012). Involvement of nucleus accumbens dopamine D1 receptors in ethanol drinking, ethanol-induced conditioned place preference, and ethanol-induced psychomotor sensitization in mice. *Psychopharmacology*.

[90] Tsai H.-C., Zhang F., Adamantidis A. (2009). Phasic firing in dopaminergic neurons is sufficient for behavioral conditioning. *Science*.

[91] Imperato A., Di Chiara G. (1986). Preferential stimulation of dopamine release in the nucleus accumbens of freely moving rats by ethanol. *Journal of Pharmacology and Experimental Therapeutics*.

[92] Wozniak K. M., Pert A., Mele A., Linnoila M. (1991). Focal application of alcohols elevates extracellular dopamine in rat brain: a microdialysis study. *Brain Research*.

[93] Adermark L., Jonsson S., Söderpalm B., Ericson M. (2013). Region-specific depression of striatal activity in Wistar rat by modest ethanol consumption over a ten-month period. *Alcohol*.

[94] Belin D., Jonkman S., Dickinson A., Robbins T. W., Everitt B. J. (2009). Parallel and interactive learning processes within the basal ganglia: relevance for the understanding of addiction. *Behavioural Brain Research*.

[95] Robertson G. S., Staines W. A. (1994). D_1_ dopamine receptor agonist-induced fos-like immunoreactivity occurs in basal forebrain and mesopontine tegmentum cholinergic neurons and striatal neurons immunoreactive for neuropeptide Y. *Neuroscience*.

[96] Hersch S. M., Ciliax B. J., Gutekunst C.-A. (1995). Electron microscopic analysis of D1 and D2 dopamine receptor proteins in the dorsal striatum and their synaptic relationships with motor corticostriatal afferents. *The Journal of Neuroscience*.

[97] Matamales M., Bertran-Gonzalez J., Salomon L. (2009). Striatal medium-sized spiny neurons: identification by nuclear staining and study of neuronal subpopulations in BAC transgenic mice. *PLoS ONE*.

[98] Centonze D., Bracci E., Pisani A., Gubellini P., Bernardi G., Calabresi P. (2002). Activation of dopamine D1-like receptors excites LTS interneurons of the striatum. *European Journal of Neuroscience*.

[99] Berlanga M. L., Simpson T. K., Alcantara A. A. (2005). Dopamine D5 receptor localization on cholinergic neurons of the rat forebrain and diencephalon: a potential neuroanatomical substrate involved in mediating dopaminergic influences on acetylcholine release. *Journal of Comparative Neurology*.

[100] Centonze D., Grande C., Usiello A. (2003). Receptor subtypes involved in the presynaptic and postsynaptic actions of dopamine on striatal interneurons. *The Journal of Neuroscience*.

[101] Rivera A., Alberti I., Martín A. B., Narváez J. A., De la Calle A., Moratalla R. (2002). Molecular phenotype of rat striatal neurons expressing the dopamine D5 receptor subtype. *European Journal of Neuroscience*.

[102] Delle Donne K. T., Sesack S. R., Pickel V. M. (1997). Ultrastructural immunocytochemical localization of the dopamine D_2_ receptor within GABAergic neurons of the rat striatum. *Brain Research*.

[103] Alcantara A. A., Chen V., Herring B. E., Mendenhall J. M., Berlanga M. L. (2003). Localization of dopamine D2 receptors on cholinergic interneurons of the dorsal striatum and nucleus accumbens of the rat. *Brain Research*.

[104] Sesack S. R., Aoki C., Pickel V. M. (1994). Ultrastructural localization of D2 receptor-like immunoreactivity in midbrain dopamine neurons and their striatal targets. *Journal of Neuroscience*.

[105] Wang H., Pickel V. M. (2002). Dopamine D2 receptors are present in prefrontal cortical afferents and their targets in patches of the rat caudate-putamen nucleus. *Journal of Comparative Neurology*.

[106] Mizuno T., Schmauss C., Rayport S. (2007). Distinct roles of presynaptic dopamine receptors in the differential modulation of the intrinsic synapses of medium-spiny neurons in the nucleus accumbens. *BMC Neuroscience*.

[107] Rivera A., Trías S., Peñafiel A. (2003). Expression of D4 dopamine receptors in striatonigral and striatopallidal neurons in the rat striatum. *Brain Research*.

[108] Wiltschko A. B., Pettibone J. R., Berke J. D. (2010). Opposite effects of stimulant and antipsychotic drugs on striatal fast-spiking interneurons. *Neuropsychopharmacology*.

[109] Sun N., Laviolette S. R. (2014). Dopamine receptor blockade modulates the rewarding and aversive properties of nicotine via dissociable neuronal activity patterns in the nucleus accumbens. *Neuropsychopharmacology*.

[110] Bracci E., Centonze D., Bernardi G., Calabresi P. (2002). Dopamine excites fast-spiking interneurons in the striatum. *Journal of Neurophysiology*.

[111] Winters B. D., Krüger J. M., Huang X. (2012). Cannabinoid receptor 1-expressing neurons in the nucleus accumbens. *Proceedings of the National Academy of Sciences of the United States of America*.

[112] Prosperetti C., Di Giovanni G., Stefani A., Möller J. C., Galati S. (2013). Acute nigro-striatal blockade alters cortico-striatal encoding: an in vivo electrophysiological study. *Experimental Neurology*.

[113] Fino E., Glowinski J., Venance L. (2007). Effects of acute dopamine depletion on the electrophysiological properties of striatal neurons. *Neuroscience Research*.

[114] Salin P., López I. P., Kachidian P. (2009). Changes to interneuron-driven striatal microcircuits in a rat model of Parkinson's disease. *Neurobiology of Disease*.

[115] Kerkerian L., Bosler O., Pelletier G., Nieoullon A. (1986). Striatal neuropeptide Y neurones are under the influence of the nigrostriatal dopaminergic pathway: immunohistochemical evidence. *Neuroscience Letters*.

[116] Vuillet J., Kerkerian L., Salin P., Nieoullon A. (1989). Ultrastructural features of NPY-containing neurons in the rat striatum. *Brain Research*.

[117] Kubota Y., Inagaki S., Kito S. (1988). Neuropeptide Y-immunoreactive neurons receive synaptic inputs from dopaminergic axon terminals in the rat neostriatum. *Brain Research*.

[118] Sørensen G., Jensen M., Weikop P. (2012). Neuropeptide Y Y5 receptor antagonism attenuates cocaine-induced effects in mice. *Psychopharmacology*.

[119] Ma Y., Zhan M., OuYang L. (2014). The effects of unilateral 6-OHDA lesion in medial forebrain bundle on the motor, cognitive dysfunctions and vulnerability of different striatal interneuron types in rats. *Behavioural Brain Research*.

[120] Salin P., Kerkerian L., Nieoullon A. (1990). Expression of neuropeptide Y immunoreactivity in the rat nucleus accumbens is under the influence of the dopaminergic mesencephalic pathway. *Experimental Brain Research*.

[121] Horner K. A., Westwood S. C., Hanson G. R., Keefe K. A. (2006). Multiple high doses of methamphetamine increase the number of preproneuropeptide Y mRNA-expressing neurons in the striatum of rat via a dopamine D1 receptor-dependent mechanism. *The Journal of Pharmacology and Experimental Therapeutics*.

[122] Dehorter N., Guigoni C., Lopez C. (2009). Dopamine-deprived striatal GABAergic interneurons burst and generate repetitive gigantic IPSCs in medium spiny neurons. *Journal of Neuroscience*.

[123] Busceti C. L., Bucci D., Molinaro G. (2012). Lack or inhibition of dopaminergic stimulation induces a development increase of striatal tyrosine hydroxylase-positive interneurons. *PLoS ONE*.

[124] Adermark L., Clarke R. B. C., Ericson M., Söderpalm B. (2011). Subregion-specific modulation of excitatory input and dopaminergic output in the striatum by tonically activated glycine and GABA_A_ receptors. *Frontiers in Systems Neuroscience*.

[125] Rahman S., McBride W. J. (2002). Involvement of GABA and cholinergic receptors in the nucleus accumbens on feedback control of somatodendritic dopamine release in the ventral tegmental area. *Journal of Neurochemistry*.

[126] Yan Q.-S. (1999). Focal bicuculline increases extracellular dopamine concentration in the nucleus accumbens of freely moving rats as measured by in vivo microdialysis. *European Journal of Pharmacology*.

[127] Pitman K. A., Puil E., Borgland S. L. (2014). GABAB modulation of dopamine release in the nucleus accumbens core. *European Journal of Neuroscience*.

[128] Chuhma N., Mingote S., Moore H., Rayport S. (2014). Dopamine neurons control striatal cholinergic neurons via regionally heterogeneous dopamine and glutamate signaling. *Neuron*.

[129] Wieland S., Du D., Oswald M. J., Parlato R., Köhr G., Kelsch W. (2014). Phasic dopaminergic activity exerts fast control of cholinergic interneuron firing via sequential NMDA, D2, and D1 receptor activation. *The Journal of Neuroscience*.

[130] Straub C., Tritsch N. X., Hagan N. A., Gu C., Sabatini B. L. (2014). Multiphasic modulation of cholinergic interneurons by nigrostriatal afferents. *Journal of Neuroscience*.

[131] Calabresi P., Centonze D., Gubellini P., Pisani A., Bernardi G. (2000). Acetylcholine-mediated modulation of striatal function. *Trends in Neurosciences*.

[132] Di Chiara G., Morelli M., Consolo S. (1994). Modulatory functions of neurotransmitters in the striatum: ACh/dopamine/NMDA interactions. *Trends in Neurosciences*.

[133] Berlanga M. L., Olsen C. M., Chen V. (2003). Cholinergic interneurons of the nucleus accumbens and dorsal striatum are activated by the self-administration of cocaine. *Neuroscience*.

[134] Laplante F., Lappi D. A., Sullivan R. M. (2011). Cholinergic depletion in the nucleus accumbens: effects on amphetamine response and sensorimotor gating. *Progress in Neuro-Psychopharmacology and Biological Psychiatry*.

[135] Hikida T., Kaneko S., Isobe T. (2001). Increased sensitivity to cocaine by cholinergic cell ablation in nucleus accumbens. *Proceedings of the National Academy of Sciences of the United States of America*.

[136] Pisani A., Bonsi P., Centonze D., Calabresi P., Bernardi G. (2000). Activation of D2-like dopamine receptors reduces synaptic inputs to striatal cholinergic interneurons. *Journal of Neuroscience*.

[137] Abercrombie E. D., DeBoer P. (1997). Substantia nigra D1 receptors and stimulation of striatal cholinergic interneurons by dopamine: a proposed circuit mechanism. *Journal of Neuroscience*.

[138] Löschmann P.-A., De Groote C., Albrecht C. (2001). [^3^H]acetycholine release in rat striatal slices is not subject to dopamine heteroreceptor supersensitivity 30 months after 6-hydroxydopamine lesion of the substantia nigra. *Naunyn-Schmiedeberg's Archives of Pharmacology*.

[139] Exley R., Cragg S. J. (2008). Presynaptic nicotinic receptors: a dynamic and diverse cholinergic filter of striatal dopamine neurotransmission. *British Journal of Pharmacology*.

[140] Threlfell S., Clements M. A., Khodai T. (2010). Striatal muscarinic receptors promote activity dependence of dopamine transmission via distinct receptor subtypes on cholinergic interneurons in ventral versus dorsal striatum. *Journal of Neuroscience*.

[141] Threlfell S., Lalic T., Platt N. J., Jennings K. A., Deisseroth K., Cragg S. J. (2012). Striatal dopamine release is triggered by synchronized activity in cholinergic interneurons. *Neuron*.

[142] Cachope R., Mateo Y., Mathur B. N. (2012). Selective activation of cholinergic interneurons enhances accumbal phasic dopamine release: Setting the tone for reward processing. *Cell Reports*.

[143] Higley M. J., Gittis A. H., Oldenburg I. A. (2011). Cholinergic interneurons mediate fast VGluT3-dependent glutamatergic transmission in the striatum. *PLoS ONE*.

[144] Rice M. E., Cragg S. J. (2004). Nicotine amplifies reward-related dopamine signals in striatum. *Nature Neuroscience*.

[145] Zhou F.-M., Liang Y., Dani J. A. (2001). Endogenous nicotinic cholinergic activity regulates dopamine release in the striatum. *Nature Neuroscience*.

[146] Exley R., McIntosh J. M., Marks M. J., Maskos U., Cragg S. J. (2012). Striatal *α*5 nicotinic receptor subunit regulates dopamine transmission in dorsal striatum. *The Journal of Neuroscience*.

[147] Burkhardt J. M., Adermark L. (2014). Locus of onset and subpopulation specificity of *in vivo* ethanol effect in the reciprocal ventral tegmental area-nucleus accumbens circuit. *Neurochemistry International*.

[148] Costa L. G., Guizzetti M. (1999). Muscarinic cholinergic receptor signal transduction as a potential target for the developmental neurotoxicity of ethanol. *Biochemical Pharmacology*.

[149] Dopico A. M., Lovinger D. M. (2009). Acute alcohol action and desensitization of ligand-gated ion channels. *Pharmacological Reviews*.

[150] Herring B. E., Mayfield R. D., Camp M. C., Alcantara A. A. (2004). Ethanol-induced Fos immunoreactivity in the extended amygdala and hypothalamus of the rat brain: focus on cholinergic interneurons of the nucleus accumbens. *Alcoholism: Clinical and Experimental Research*.

[151] Pereira P. A., Neves J., Vilela M., Sousa S., Cruz C., Madeira M. D. (2014). Chronic alcohol consumption leads to neurochemical changes in the nucleus accumbens that are not fully reversed by withdrawal. *Neurotoxicology and Teratology*.

[152] Nestby P., Vanderschuren L. J. M. J., De Vries T. J. (1999). Unrestricted free-choice ethanol self-administration in rats causes long-term neuroadaptations in the nucleus accumbens and caudate putamen. *Psychopharmacology*.

[153] Larsson A., Edström L., Svensson L., Söderpalm B., Engel J. A. (2005). Voluntary ethanol intake increases extracellular acetylcholine levels in the ventral tegmental area in the rat. *Alcohol and Alcoholism*.

[154] Russell V. A., Lamm M. C. L., Taljaard J. J. F. (1988). Effect of ethanol on [^3^H]Dopamine release in rat nucleus accumbens and striatal slices. *Neurochemical Research*.

[155] Adermark L., Clarke R. B. C., Söderpalm B., Ericson M. (2011). Ethanol-induced modulation of synaptic output from the dorsolateral striatum in rat is regulated by cholinergic interneurons. *Neurochemistry International*.

[156] Adermark L., Söderpalm B., Burkhardt J. M. (2014). Brain region specific modulation of ethanol-induced depression of GABAergic neurons in the brain reward system by the nicotine receptor antagonist mecamylamine. *Alcohol*.

[157] Molander A., Löf E., Stomberg R., Ericson M., Söderpalm B. (2005). Involvement of accumbal glycine receptors in the regulation of voluntary ethanol intake in the rat. *Alcoholism: Clinical and Experimental Research*.

[158] Molander A., Söderpalm B. (2005). Accumbal strychnine-sensitive glycine receptors: an access point for ethanol to the brain reward system. *Alcoholism: Clinical and Experimental Research*.

[159] Molander A., Lidö H. H., Löf E., Ericson M., Söderpalm B. (2007). The glycine reuptake inhibitor org 25935 decreases ethanol intake and preference in male Wistar rats. *Alcohol and Alcoholism*.

[160] Darstein M., Landwehrmeyer G. B., Kling C., Becker C.-M., Feuerstein T. J. (2000). Strychnine-sensitive glycine receptors in rat caudatoputamen are expressed by cholinergic interneurons. *Neuroscience*.

[161] Darstein M., Löschmann P. A., Knörle R., Feuerstein T. J. (1997). Strychnine-sensitive glycine receptors inducing [3H]-acetylcholine release in rat caudatoputamen: a new site of action of ethanol?. *Naunyn-Schmiedeberg's Archives of Pharmacology*.

[162] Waldvogel H. J., Baer K., Allen K. L., Rees M. I., Faull R. L. M. (2007). Glycine receptors in the striatum, globus pallidus, and substantia nigra of the human brain: an immunohistochemical study. *Journal of Comparative Neurology*.

[163] Borkar C. D., Upadhya M., Shelkar G. P., Subhedar N., Kokare D. M. (2015). Neuropeptide Y system in accumbens shell mediates ethanol self-administration in posterior ventral tegmental area. *Addiction Biology*.

